# Innate immune system: the no man’s land where discover new biomarkers for gluten-related-disorders 

**Published:** 2015

**Authors:** Roberto Assandri, Marta Monari, Anna Colombo, Alessandro Montanelli

**Affiliations:** *Humanitas Clinical and Research Center, Rozzano, Milan, Italy*

Celiac disease (CD) is now considered, more than a just gluten sensitivity enteropathy, a multiple and systemic immune-mediate disorder triggered by the ingestion of wheat gluten and related proteins. Following the discovery of a link between gluten and CD, it was demonstrated that gliadin, one of the two principal protein groups comprising gluten, plays a key role in CD pathogenesis. It has since become clear that the different and crucial roles of gliadin in CD result from its ability to activate multiple signalling pathways that modulate CD pathology and CD progression (1,2). 

It is well known that CD is strongly associated with specific human leukocyte antigen (HLA) class II genes, known as HLA-DQ2 and HLA-DQ8, located on chromosome 6p21 (3). 

Gliadin-specific T-cell responses have been found to be enhanced by the action of intestinal transglutaminases. The infiltration of T cells in the lamina propria of the active celiac lesion is dominated by CD4+ memory T cells (CD45RO+) bearing the α/β T-cell receptor (TCR) (4). 

The adaptive immune response is initiated by APCs, primarily dendritic cells (DCs) but also macrophages and B cell subsets, which present to T-cell antigenic fragments in complex with cell surface MHC class II molecules (4).

However, it is now known that to induce sufficient adaptive immune responses, antigens have to be recognized in the context of an activated innate immune system (5).

Activation of innate immunity has been associated with the presence of toxic p31–49 gliadin peptides. In epithelial cells (ECs) toxic glaidin peptides are internalized into early endosomes and lysosomial enzymes or cytosolic enzymes can degrade late endosomes and the peptides. In celiac disease, toxic p31–49 peptide accumulates in the early endosome (6). This event was found associated to an over reactive oxygen species (ROS) and transglutaminase 2 (TG2) production, which may cause a decrease of peroxisome proliferator-activated receptors (PPARs) and a consequent activation of the NF-kB pathway (7).

Breaking immunological tolerance, gliadin peptides stimulate different type of cell, particularly dendritic cells (DCs), which results in leukocyte infiltration and inflammation of gut mucosa (8). Given their high plasticity and maturation ability in response to local danger signals derived from innate immunity, dendritic cells are key actors in the connection between innate immunity and adaptive immunity responses (8). 

In addition to their role as sentinels DCs also act as immune system sensors given their high expression of pattern recognition receptors, including Toll-like receptors (TLRs) (11). 

TLRs comprise a large family of the pathogen-pattern recognition receptors (PPRR) originally identified in *Drosophila *in the mid-1990s as a *Toll protein *(9).

Some studies have provided important clues about the mechanisms of TLR-mediated control of adaptive immunity orchestrated by dendritic cell populations in distinct anatomical locations (10).

However, an increasing number of reports show a more diverse expression of TLRs, including epithelial cells (11). 

TLR4 is one of the best characterized and the first member of the TLR family to be discovered as a PPRR. TLR4 signalling is implicated in the innate immune responses against a wide-range of microbes, including gram-negative and -positive bacteria, mycobacteria, spirochetes, yeasts, and some viruses and mammary tumour viruses (12). 

TLR4 is implicated in a diverse range of pathological processes associated with autoimmune diseases such as psoriasis, diabetic retinopathy, thrombosis, and inflammatory disorders including arthritis and atherosclerosis (13).

Recently some finding suggested that the TLR4 are involved in pathogenesis of CD and Non-Celiac Gluten Sensitivity (NCGS) (14). However, in contrast to CD, the pathogenesis of NCGS is still poorly defined. Enhanced neutrophil recruitment, gut and mucosal inflammation, changes in intestinal microbiota and immune response to gliadin are features common to NCGS and CD (15).

In contrast to CD, small-intestine expression TLR4 and generally of innate immunity is greater in patients with NCGS (14). 

In a very recent work Jelinkova et al. defined a hypothetical signalling pathways related to innate immunity that involved TLR4, NF-kB and inflammasome in the pathogenesis of gluten sensitivity (16). 

Furthermore, Junker et al. demonstrated that mice deficient in TRL4 signalling were protected from intestinal and systemic immune response to gluten or to strong activation of innate immunity system (17). 

In the recent years the long pentraxin PTX3 are characterized as a key component of humoral pattern and innate immune system. Pentraxins are a family of multimeric proteins that were divided into short and long pentraxin, based on the primary structure: C-reactive protein (CRP) is the prototype of the short pentraxin subfamily while pentraxin 3 (PTX3) is the prototypic long pentraxin. (13). Cells involved in innate immunity such as polymorphonuclear leukocytes, macrophages, and dendritic cells, produce PTX3 (18-21).

The corresponding PTX3 human gene is located on chromosome 3 band q 25. The proximal promoter shares numerous transcription factor binding sequences (Pu-1, AP1, NF- kB, SP1 and NF-IL6) (19). It has been shown that NF-kB binding site is essential for transcriptional response (22). In contrast to the IL-6-mediated CRP production, PTX3 is produced in response to several primary inflammatory signals such as TLR agonists, IL-1 and tumour necrosis factor alpha (TNF-alpha) (23).

Under the influence of TNF-alpha and IL-1, ECs are able to produce considerable amounts of PTX3 (19,20). It was elegantly demonstrated that in the intestinal mucosa and corresponding ECs, PTX3 was strictly necessary for NF-kB activation in model of intestinal reperfusion injury and underlined a fundamental role of PTX3 in mediating tissue inflammation under sterile conditions (22).

A recent Chinese study also showed that *Lactobacillus acidophilus *could transiently regulate the immunity and inflammatory mediator factor PTX3 expression through rapidly activating NF-kB signalling pathway in intestinal epithelial cells (CaCo-2 cells) (23).

In a recent study Chen et al. clearly demonstrated that endothelial PTX3 plays a pivotal role in the pathogenesis of ischemic acute kidney injury, via TLR4 and reactive oxygen species (24).

In a very recent study we demonstrated that PTX3 serum level was high in patients with active disease patients in different intestinal mucosa condition (Marsh 1-3 histological grade). Furthermore, PTX3 serum concentration was significantly low in GFD patients than in active CD patients with mucosal damage. These evidences suggested that PTX3 concentration could reflect disease activity. The GFD and consequently the absence of toxin gliadin peptides could represent the “silencer” element of PTX3 expression. Yet the levels of PTX3 correlated with the DGP-IgA levels but not with the tTG IgA levels or with serum CRP. In addition, PTX3 serum levels correlated with the major extraintestinal manifestation such as anaemia and lower iron concentration (25). 

We also provided new evidences showing that after a strict compliance of GFD, PTX3 serum levels could follow the improvement of both gastrointestinal and extraintestinal symptoms. 

In the same work we demonstrated a linear correlation between DGP-IgA levels and PTX3 serum levels (25). This evidence acquired some interest if we considered that many patients with NCGS showed elevated prevalence “first generation” IgG class antibodies against native gliadin peptides (AGA-IgG) (15, 26-28). It is also evident that a common “wooden horse” of CD and NCGS is the ingestion of gluten and related toxic peptides.

We suggested an interesting hypothesis in which PTX3 could be ever overexpress during gliadin and gluten exposure and modulate intestinal immune response to gluten and relate proteins by the pathway that involve NF-kB and TLR4. In this interesting scenario we certainly advanced the hypothesis that PTX3 could perform a critical role as mediator of inflammation in several steps that link toxic glaidin ingestion and tissue damage.

At the moment we don’t have adequate elements to suggest the use of PTX3 in diagnosis of NCGS, but we are obliged to speculate about the possible role of PTX3 molecules in NCGS pathogenesis. PTX3 is an exploring molecule in the “No man’s land” of NCGS biomarkers.
